# Accuracy of acetabular cup positioning using imageless navigation

**DOI:** 10.1186/1749-799X-6-40

**Published:** 2011-08-10

**Authors:** Erik Hohmann, Adam Bryant, Kevin Tetsworth

**Affiliations:** 1 Musculoskeletal Research Unit, CQ University, Yaamba Road, Rockhampton 4700, Australia; 2 Department of Orthopaedic Surgery, Rockhampton Hospital, Canning Street, Rockhampton QLD 4700, Australia; 3 Centre for Health, Exercise and Sports Medicine, Faculty of Medicine, The University of Melbourne, 200 Berkeley Street, Melbourne VIC 3010, Australia; 4 Department of Orthopaedic Surgery, Royal Brisbane Hospital, Butterfield Street, Herston QLD 4029, Australia; 5 CONROD Professor of Orthopaedic Trauma Surgery, Division of Surgery, University of Queensland Medical School, Butterfield Street, Herston QLD 4029

## Abstract

**Background:**

Correct placement of the acetabular cup is a crucial step in total hip replacement to achieve a satisfactory result and remains a challenge with free-hand techniques. Imageless navigation may provide a viable alternative to free-hand technique and improve placement significantly. The purpose of this project was to assess and validate intra-operative placement values for both inclination and anteversion as displayed by an imageless navigation system to post-operative measurement of cup position using high resolution CT scans.

**Methods:**

Thirty-two subjects who underwent primary hip joint arthroplasty using imageless navigation were included. The average age was 66.5 years (range 32-87). 23 non-cemented and 9 cemented acetabular cups were implanted. The desired position for the cup was 45 degrees of inversion and 15 degrees of anteversion. A pelvic CT scan using a multi-slice CT was used to assess the position of the cup radiographically.

**Results:**

Two subjects were excluded because of dislodgement of the tracking pin. Pearson correlation revealed a strong and significant correlation (r = 0.68; p < 0.006) for cup inclination and a moderate non-significant correlation (r = 0.53; p = 0.45) between intra-operative readings and cup placement for anteversion.

**Conclusions:**

These findings can be explained with the possible introduction of systematic error. Even though the acquisition of anatomic landmarks is simple, they must be acquired with great precision. An error of 1 cm can result in a mean anteversion error of 6 degrees and inclination error of 2.5 degrees. Whilst computer assisted surgery results in highly accurate cup placements for inclination, anteversion of the cup cannot be determined accurately.

## Background

Correct placement of the acetabular cup in total hip arthroplasty is a crucial step to achieve a satisfactory result and remains a challenge with free-hand techniques [1-3]. Indeed, malpositioning can induce early loosening, high wear and postoperative dislocation [4-6]. Various investigators have demonstrated that conventional free-hand positioning can result in a high percentage of unacceptable acetabular cup placements [2,3,7,8].

Imageless navigation may provide a viable alternative to free-hand techniques and the use of mechanical guides and may improve placement significantly [7,9,10]. Previous authors have demonstrated that cup alignment significantly improved with the use of computer navigation [3,9,11-14]. Imageless computer aided navigation relies on a pelvic coordinate system which uses bony landmarks (anterior superior iliac spines and pubic tubercle) to define the anterior frontal plane [15,16]. These bony landmarks are determined by palpation and digitization through the overlying soft-tissue with a metal pointer [16]. Manual digitization can potentially cause measurement error which, in turn, can result in excessive tilt of the cup in the frontal plane. This is particularly problematic in obese patients where excess soft tissue can completely obscure bony landmarks. Clearly, the introduction of systematic error may lead to cup placement which differs from the intra-operative readings when using imageless navigation.

Therefore, the purpose of this study was to assess and validate intra-operative placement orientation as displayed by the navigation unit to post-operative measurement of cup position using high resolution CT scans. We hypothesized that inclination is highly accurate as the anterior superior iliac spines are easily palpable even in obese patients. In contrast, we hypothesized that anteversion is inaccurate due to the underlying soft-tissue and the difficulty in identifying the pubic tubercle.

## Methods

### Patient selection

Between June 2005 and December 2007, 32 patients underwent primary hip joint replacement using imageless navigation. Two patients had to be excluded because of intra-operative dislodgement of the tracking pin. The mean patient age was 66.5 ± 14 (range 28-87) years. There were 16 males (mean age 62.2 ± 12.2) and 14 females (mean age 71.4 ± 14.7. The mean weight was 85.6 ± 14 kg (range 57-112), the mean height measured 169 ± 8.6 cm (range153-186) and the average BMI was 30.04 ± 4.6 kg/m^2 ^(range 20.9-39.5). Twenty one non-cemented and 9 cemented acetabular cups were implanted. The average size of the non-cemented cup was 53 mm (range 46-60) and averaged 54 (range 50-58) for the cemented cup. The main indication was primary osteoarthritis (n = 25), osteonecrosis (n = 5), displaced neck of femur fracture (n = 1) and failed screw fixation with head collapse after neck of femur fracture (n = 1). In 17 subjects, hip arthroplasty was performed on the right hip and 15 subjects had a left total hip arthroplasty. Surgery was performed by a single surgeon who was an experienced user of the imageless navigation system.

### Sequence of Navigation

**Figure 1:**
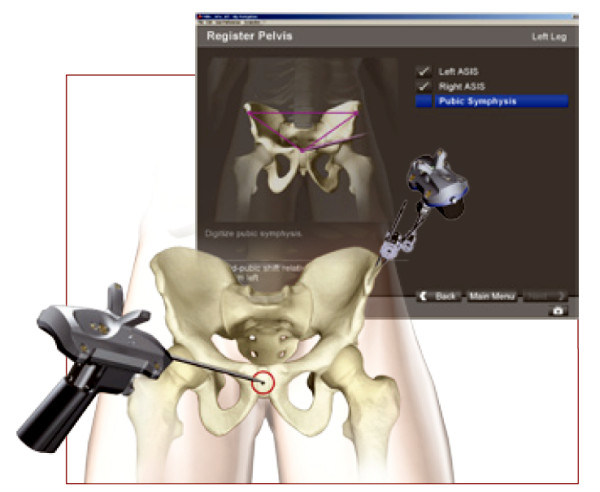
**Once the Schanz screw was inserted into the ipsilateral anterior superior iliac, spine and the pelvic navigation tracker was attached, bony landmarks (ASIS, pubic, tubercle) were determined and digitalized with a metal pointer**.

An imageless computer navigation system (Stryker^® ^Navigation System, Stryker Corporation, Kalamazoo, MI, USA) was used for all surgery. Patients were placed supine. A Schanz screw was inserted into the ipsilateral anterior superior iliac spine (ASIS) through a stab incision. The pelvic navigation tracker was attached to the screw. Bony landmarks (ASIS, pubic tubercle) were determined and digitalized with a metal pointer (Figure 1). Once the frontal plane was defined by the computer the hip was moved through arrange of motion to determine the centre of rotation. Prior to dislocation and resection of the femoral head the piriformis fossa was digitalized. The acetabular fossa and rim was then digitalized. Once the landmarks were defined, the navigation system determined inclination and anteversion of the acetabulum.

### Surgical Technique

The surgical procedures were performed using a lateral Hardinge approach in all cases. Reaming, trial cup position and final cup position was performed navigated. The aim was to achieve 45 degrees inclination and 15 degrees anteversion. Intra-operative cup position was recorded. The contemporary cup (Stryker^®^) was used with cement and the Trident cup (Stryker^®^) was used without cement.

### Postoperative CT

Post-operatively, a multi-slice CT scan was obtained on day one post surgery using a helical CT scanner (Somatom; Siemens^®^, Munich, Germany). All CT scans were performed by the same radiology technician to a pre-established protocol. Two millimeter slices were obtained in all cases. The position of the pelvis was standardized by reformatting the images to the frontal plane defined by both anterior superior iliac spines and the pubic tubercle. The largest cup diameter on the coronal plane was identified and the inclination was measured. Similar anteversion was measured by identifying the largest cup diameter on an axial plane. All measurements were performed three times and averaged.

### Statistical Analysis

To determine sample size a power calculation was performed. The study was designed to provide the number of cases required to discover a statistical significant (*p *= 0.05) correlation of r ≥ 0.50 between intra-operative cup placement and post-operative CT measurements. The sample size calculation based on these parameters indicated that 29 patients were needed to provide 90% statistical power.

Pearson's product-moment correlation coefficients were used to establish the strength of the relationships between intra-operative cup placement and post-operative CT measurements. All analyses were conducted using SPSS (Version 12.0.1; Chicago, IL) for Windows.

## Results

### Inclination

**Figure 2:**
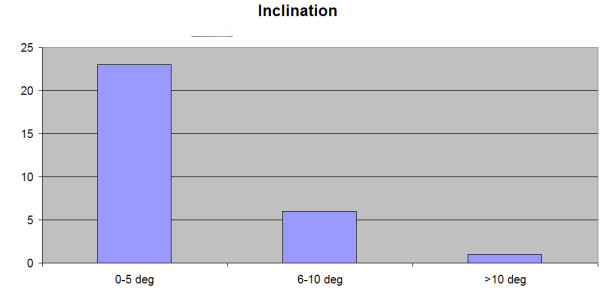
**The numbers of cups placed within 5 degrees, 6-10 degrees and more than 10 degrees of the intra-operative final cup placement inclination readings as displayed by the navigation system are shown**.

Differences between navigation-derived intra-operative final cup inclination and final CT cup inclination of all 30 cups are shown in Figure 2. In 23 subjects, cup placement was within 5 degrees of intra-operative readings. Six cups were placed within 10 degrees and one cup was placed with a difference of more than 10 degrees (Figure 2). A mean difference of 3.8^0 ^+ 3.8^0 ^(range 0°-15.7°) between intra-operative cup placement and postoperative measurement was observed. Pearson correlation revealed a strong, significant correlation (r = 0.68; *p *< 0.006) for cup inclination between intra-operative final cup placement and cup placement measured by CT.

### Anteversion

**Figure 3:**
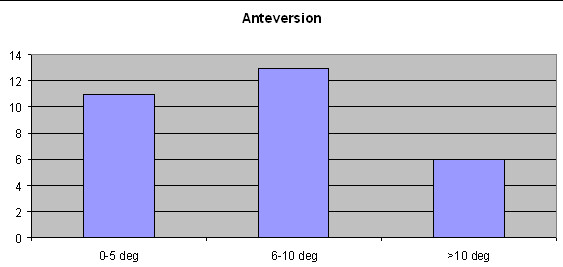
**The numbers of cups placed within 5 degrees, 6-10 degrees and more than 10 degrees of the intra-operative final cup placement anteversion readings as displayed by the navigation system are shown**.

Differences between navigation-derived intra-operative final cup anteversion and final CT cup anteversion of all 30 cups are shown in Figure 3. Only 11 cups were placed within 5 degrees of navigation unit readings. In 13 cups anteversion readings and final CT results were within 10 degrees and 6 cups were placed outside 10 degrees (Figure 3). A mean difference of 7.7^0 ^+ 7.6^0 ^(range 0°-26°) between intra-operative cup placement and post-operative measurement was observed. Pearson correlation revealed a moderate, non-significant correlation (r = 0.53; *p *= 0.45) between intra-operative readings and cup placement.

## Discussion

Imageless navigation is absolutely dependent upon accurate identification and digitization of appropriate bony landmarks. Unfortunately, anatomical landmarks are often obscured in larger patients, and may lead to corresponding difficulty in positioning an implant accurately. To assess the clinical viability of various landmarks and particular methods for imageless navigation, it is necessary to evaluate the accuracy of navigated cup position intra-operative values in comparison to the implants final position when measured objectively post-operatively. Using one specific technique for imageless navigation, our results demonstrate there was a strong (r = 0.68) and (p = 0.006) and significant relationship for inclination, but only a moderate non-significant relationship (r = 0.36) for anteversion when comparing intra-operative cup position and post-operative final implant position measurements.

These results compare favorably with those previously published by other authors.

Ybinger et al [17] observed a mean difference between navigation recorded and CT measured inclination of 3.5^0 ^degrees and a mean difference 6.5^0 ^degrees for anteversion in 37 subjects. In an earlier laboratory study with 10 cadavers, Kalteis et al [18] observed a median difference of 1.5^0 ^for inclination and 0.5^0 ^for anteversion. Fukunishi et al [19] analyzed accuracy of cup navigation in 27 total hip arthroplasties. Intra-operative cup inclination ranged from 39.9^0 ^to 46.6^0 ^degrees with a mean angle of 43.5^0 ^degrees compared to a range of 38.1^0 ^to 55.0^0 ^degrees with a mean angle of 44.9^0 ^degrees post-operative. Mean intra- and post-operative values were 11.1^0 ^(range 0-17.8) degrees and 13.5^0 ^(range 5.1-21.6) degrees respectively. A discrepancy of > 5^0 ^degrees was observed in one case. A mean difference of 1.9^0 ^degrees for inclination and 2.6^0 ^degrees for anteversion was calculated between intra- and post-operative values. Dorr et al [12] observed an accuracy of 4.4^0 ^degrees for inclination and 4.1^0 ^degrees for anteversion with no outliers greater than 5^0 ^degrees. They concluded that surgeons can trust a validated computer navigation system for cup position.

Our results compare favorably with these previous studies, although only Fununkashi et al [19] (one patient with a discrepancy of > 5^0 ^degrees) and Dorr et al [12] (no outliers) reported outliers. In contrast to the previous authors [12,17-19], we documented similar differences but we have observed more frequent cup placements with a discrepancy of > 5^0 ^degrees. It may therefore be more important to report on the numbers of outliers rather than documenting mean differences, ranges and standard deviations. This would perhaps be a better method to describe the accuracy of a navigation system more definitely.

One possible explanation for the differences between our results and other authors may be attributed to the fact that that average BMI of our cohort group is above 30. In this respect, it has been demonstrated by several authors [20-22] that the overlying soft tissue obscures bony landmarks and introduces measurement error. Ybinger et al [17] reported a positive moderate, significant (r = 0.44, *p *= 0.007) relationship between thickness of soft tissues over the ASIS and inclination as well as positive moderate, significant correlation (r = 0.52, *p *= 0.001) between soft tissues over the pubic tubercles and anteversion angles.

**Figure 4:**
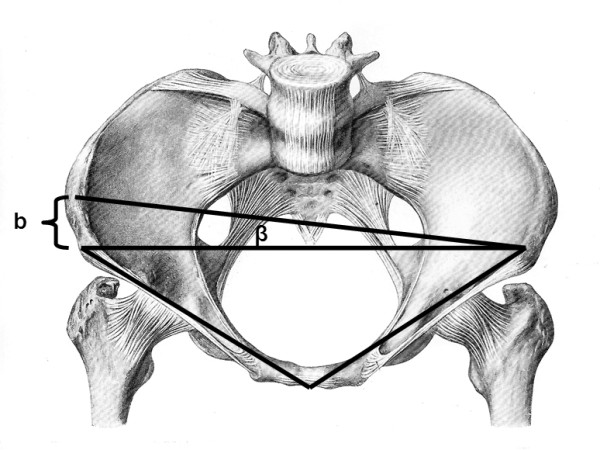
**Failure to digitize the anterior iliac spines correctly can introduce systematic error**. A difference of one centimeters ("b") between the right and left ASIS introduces an error of 2.5 and a difference of two centimeters ("b") can result in a 5 degree error for inclination.

**Figure 5:**
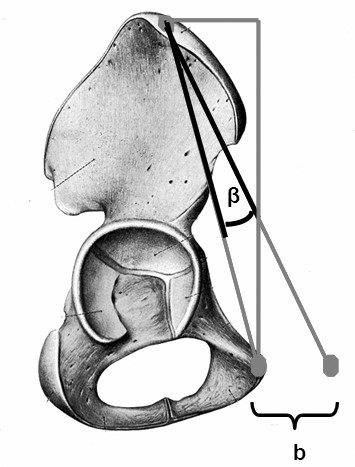
**Failure to digitize the pubic tubercle can introduce systematic error**. A difference of one centimeters ("b") too anterior or posterior of the pubic tubercle introduces an error and a difference of two centimeter ("b") can results in a 11 degree error for anteversion.

When digitizing the ASIS, measurement errors of one centimeter (left versus right) and two centimeters may introduce errors up to 2.5° and 5° degrees in cup alignment (Figure 4). Similarly, if digitization of the pubic symphysis is measured either one centimeter too anterior or posterior, a measurement error of 6° will result. A difference of 2 centimeters increases the error to 11° (Figure 5).

The difficulty in palpating the pubic symphysis in subjects with a thicker soft tissue envelope and the shorter distance between the superior aspect of the anterior pelvic triangle and the symphysis explains the higher error that we observed for anteversion. Consequently, obese patients may not be suitable for hip navigation given the increased risk of measurement error. However this criteria was not studied in this project.

## Conclusion

The results of our study suggest that there is a strong and significant correlation between intra-operative final cup placement and post-operative values for inclination and a moderate non-significant correlation for anteversion. Furthermore, we demonstrated cemented cup placement is more accurate, despite the relatively small sample size. Although the location of anatomic landmarks is simple; precision is imperative in order to reduce error. These findings are most likely due to the introduction of systematic error. Small acquisition errors can result in substantial systematic errors introduced by inadequate calculation of the anterior pelvic plane by the navigation system. The results of this study suggest that imageless navigation is a tool which is reliable, easy to use and potentially reduces the variation in free-hand placement of acetabular cups. Further work is warranted to increase the precision of cup positioning using this particular navigation system.

## Competing interests

The authors declare that they have no competing interests.

## Authors' contributions

EH: chief investigator, developed design and methods, analyzed data, drafted manuscript and is responsible for the final approval of the manuscript

AB:assisted with the design and analysis, assisted with the first draft and critically reviewed further versions, co-author who applied all statistical analysis and was involved in interpretation of results.

KT:assisted with the design and analysis, assisted with the first draft and critically reviewed further versions
